# Prevention of relapses with levamisole as adjuvant therapy in children with a first episode of idiopathic nephrotic syndrome: study protocol for a double blind, randomised placebo-controlled trial (the LEARNS study)

**DOI:** 10.1136/bmjopen-2018-027011

**Published:** 2019-08-01

**Authors:** Floor Veltkamp, Djera H Khan, Christa Reefman, Susan Veissi, Hedy A van Oers, Elena Levtchenko, Ron A A Mathôt, Sandrine Florquin, Joanna A E van Wijk, Michiel F Schreuder, Lotte Haverman, Antonia H M Bouts

**Affiliations:** 1 Paediatric Nephrology, Amsterdam UMC, University of Amsterdam, Amsterdam, The Netherlands; 2 Pathology, Amsterdam UMC, University of Amsterdam, Amsterdam, The Netherlands; 3 Paediatric Nephrology, Radboudumc, Nijmegen, The Netherlands; 4 Psychosocial Department, Amsterdam UMC, University of Amsterdam, Amsterdam, The Netherlands; 5 Paediatric Nephrology, Universitaire Ziekenhuizen Leuven, Leuven, Belgium; 6 Hospital Pharmacy, Amsterdam UMC, University of Amsterdam, Amsterdam, The Netherlands; 7 Paediatric Nephrology, Amsterdam UMC, Vrije Universiteit Amsterdam, Amsterdam, The Netherlands

**Keywords:** idiopathic nephrotic syndrome, paediatric nephrology, levamisole, randomised controlled trial, quality of life

## Abstract

**Introduction:**

Idiopathic nephrotic syndrome (INS) is characterised by a high relapse rate up to 80% after initial response to standard therapy with corticosteroids. Steroid toxicity is common and causes a great burden of disease that negatively influences the health-related quality of life (HRQoL). Recently, studies have shown that levamisole, an anthelminthic drug, significantly improves relapse-free survival in children with frequent relapses or steroid dependency. Compared with other steroid-sparing drugs, levamisole has relatively few side effects. We hypothesise that adding levamisole to standard therapy with corticosteroids in children with a first episode of INS will prevent relapses, decrease cumulative dosage of steroids used and improve HRQoL. This paper presents the study protocol for the LEARNS study (LEvamisole as Adjuvant therapy to Reduce relapses of Nephrotic Syndrome).

**Methods and analysis:**

An international, double-blind, placebo-controlled randomised trial will be conducted in 20 participating hospitals in the Netherlands and Belgium. Participants (n=92) with a first episode of INS, aged 2–16 years, who achieve remission after 4 weeks of oral prednisolone will be randomly assigned (1:1) to receive either levamisole 2.5 mg/kg alternate day or placebo added to prednisolone (18-week tapering schedule) for a total of 24 weeks. Follow-up will be until 2 years after first presentation. Additionally, parents and/or children will fill out five HRQoL questionnaires. Primary outcome of the LEARNS study is occurrence of relapses within 12 months after first presentation. Secondary outcomes include time to first relapse, cumulative steroid dose after 2 years, safety parameters and quality of life scores.

**Ethics and dissemination:**

The trial was approved by the Medical Ethical Committee. Results of the study will be published in a peer-reviewed journal.

**Trial registration number:**

NL6826, 2017-001025-41

**Strengths and limitations of this study**

- Investigator-initiated, multicentre, double-blind, randomised, placebo-controlled study.
- Additional study on health-related quality of life.
- Homogenous patient population.
- Possibility to pool data with similar ongoing trial (Nephrovir-3).
- Sample size only powered on primary outcome (ie, not for subgroup analyses).

## Background

Idiopathic nephrotic syndrome (INS) is a rare disease that predominantly affects young children. In the Netherlands, the yearly incidence is 1.52 per 100 000 children (an estimated 60 new cases per year).^1^ INS is characterised by proteinuria, hypoalbuminaemia and oedema. It is often accompanied by hyperlipidaemia. Initial treatment consists of 8–20 weeks of corticosteroids, of which at least 4 weeks single-daily dose of 60 mg/m^2^ followed by an alternate schedule.^2^ The vast majority of patients shows remission within 4 weeks, after which the diagnosis minimal change disease is assumed and no kidney biopsy is indicated.^3^ Unfortunately, 80% of the responders experience at least one relapse; half of those patients experiencing frequent relapses (FRNS) or becoming steroid-dependent (SDNS).^4^ Relapses are further treated with steroids; however, steroids are associated with many side effects.^5^ In these cases, steroid-sparing drugs are recommended.^2^ These include cyclophosphamide, calcineurin inhibitors, mycophenolate mofetil (MMF) and rituximab. But still, all come with substantial toxicity. A promising steroid-sparing agent is levamisole, an anthelminthic drug mostly used in a veterinary setting. Levamisole is, contrary to other steroid-sparing drugs, not an immunosuppressant, but acts as an immunomodulator. It has relatively few side effects of which neutropenia is the most serious and most common that requires frequent check-ups. Recent trials show a beneficial effect of adjuvant treatment with levamisole to corticosteroids in children with FRNS or SDNS, but also that treatment is safe.^6–8^

In order to prevent the frequency of relapses, several steroid schedules for the treatment of the first episode of INS have been studied, for instance, comparing a longer with a shorter course.^4^ To our knowledge, no trials have been performed investigating the efficacy of levamisole as an adjuvant agent to initial steroid treatment on preventing relapses. Currently, a study similar to ours is underway in France (Nephrovir-3).

We hypothesise that adding levamisole to standard steroid treatment in children with a first episode of INS will prevent relapses. This is substantiated by the fact that (1) INS is characterised by a T helper cell (Th) type 2 (antibody-mediated) immune response, and that (2) levamisole is able to skew the Th type 2 response into a Th type 1 (cell-mediated) response,^9 10^ and thus, restoring the balance. The aim of the LEARNS study is to investigate the efficacy and safety of adjuvant levamisole in children with a first episode of INS by conducting a randomised, double-blind, placebo-controlled clinical trial in 20 hospitals in the Netherlands and Belgium. The trial is extended with a sub-study on the health-related quality of life (HRQoL).

## Methods and analysis

### Study design

**Figure 1:**
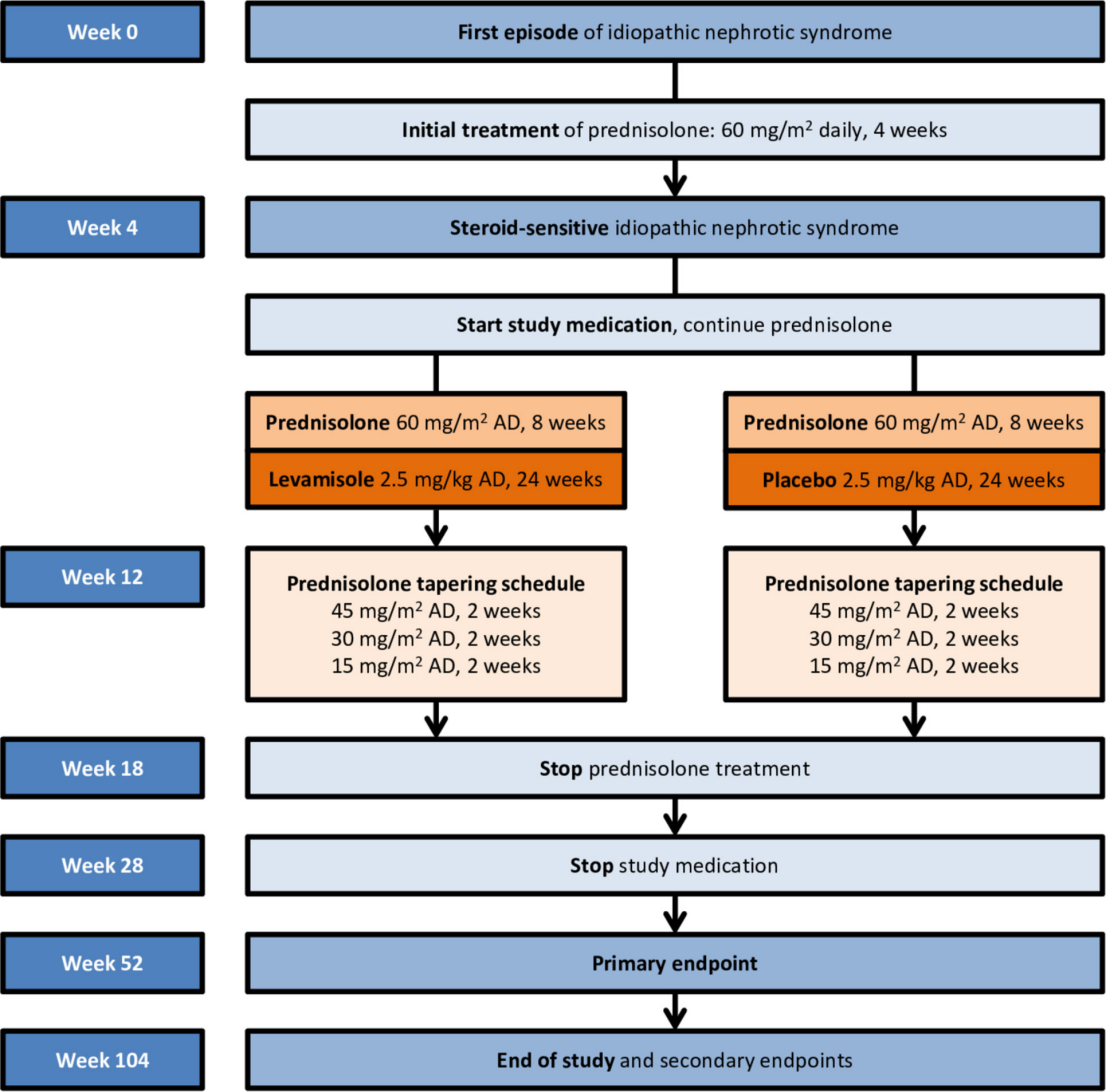
Schematic overview of study design. AD, alternate days.

The LEARNS study is an international, phase 3, double-blind, parallel, two-arm, randomised, placebo-controlled superiority trial. The study is coordinated by the Amsterdam University Medical Centers, University of Amsterdam and is conducted in 15 and 5 participating centres in the Netherlands and Belgium, respectively. Local investigators are paediatric nephrologists or paediatricians with a special interest in paediatric nephrology in both university and local hospitals. Together, the participating sites cover a large geographical area. The expected total duration of the study is 4 years, consisting of 2 years of inclusion and 2 years of follow-up since first presentation (figure 1).

### Objectives

The primary objective of the study is to investigate the effect of additional levamisole to standard steroid therapy in comparison with placebo from 4 to 28 weeks (figure 1) after the start of the first episode of steroid-sensitive INS (SSNS) in children (age 2–16 years) on the occurrence of any relapse within 12 months after first presentation.

Secondary objectives are:

- To investigate differences between the two study groups in time to first relapse, the number of relapses, cumulative steroid dosage and the occurrence of SDNS and FRNS in a 2-year period.
- To investigate HRQoL, psychosocial adjustment, parental distress at different stages of treatment (longitudinal and cross-sectional) and to identify medical and personal factors related to HRQoL.
- To investigate the consequences of the disease on days of missing school for children, (financial) problems for parents, outpatient visits and hospital admissions.

### Population

Children between 2 and 16 years of age who present with a first episode of INS will be screened for eligibility. Those who achieve remission within 4 weeks of oral treatment with corticosteroids will be screened for inclusion in the randomised controlled trial (RCT). Remission is defined as the absence of proteinuria for three consecutive days, confirmed by quantitative urinalysis (protein–creatinine ratio <20 mg/mmol creatinine). All inclusion and exclusion criteria are described in box 1. SSNS will be defined according to current guidelines.^2^

Box 1 **Inclusion and exclusion criteria for participation in the randomised controlled trial**

**Inclusion criteria**

First episode of steroid-sensitive INS, as defined by:

- Proteinuria (>200 mg/mmol creatinine).
- Hypoalbuminaemia (<25 g/L).
- Oedema.

Remission, defined by:

- Absence of proteinuria (<20 mg/mmol creatinine) for three consecutive days within 4 weeks of steroid treatment.

Complement C3 within normal range.

Age 2–16 years.

Written informed consent.

Weight >9 kg.

Ability to swallow 5 mg (placebo) tablet (successful swallowing test in children<6 years).

Negative pregnancy test in girls who are of childbearing potential.

Absence of contraindication for levamisole use, defined as neutropenia (<1500/mm^3^).

**Exclusion criteria**

Previous episode(s) of INS.

Steroid-resistant INS, defined by:

- Persistent proteinuria at 4 weeks after start steroid treatment.

Previous or current malignancy, diabetes mellitus, current liver disease or epileptic convulsions.

Hypersensitivity to levamisole or one of its substances (lactose).

Inability to comply with the study protocol.

### Intervention

After confirming the diagnosis of INS, all patients will be treated with oral prednisolone according to the French protocol, which is an 18-week tapering schedule (figure 1).^11^ In addition to prednisolone, all patients will receive vitamin D (400 IE/day for children <12 years; 800 IE/day for children ≥12 years). When participating in the trial, concomitant use of any immunosuppressive and/or stimulant medication other than prednisolone is prohibited. Because of their anti-proteinuric actions, non-steroid anti-inflammatory drugs, ACE-inhibitors and angiotensin II-receptor blockers are prohibited as well. If remission is achieved at week 4, study subjects will receive 2.5 mg/kg levamisole or placebo on alternate days for a total of 24 weeks (figure 1). The current dosage schedule has been established in a previous study.^12^ The dose of the study medication is calculated based on the patient’s weight, with a maximum of 150 mg/dose. Prednisolone and study medication need to be taken simultaneously. A missed dose can be taken within 24 hours of scheduled time. After 24 hours or more, the dose will not be taken and the next dose will be as scheduled. Study treatment will be (temporarily) stopped in case of the following situations: relapse, neutropenia <500/mm^3^, elevated liver enzymes >5× upper limit of normal, at the patient’s and/or their parent’s request, and/or withdrawal of informed consent. More details of temporarily stopping the study medication in case of moderate to mild neutropenia can be found in the online supplement 1. In case of a relapse, treatment will be determined by the treating physician, but generally consists of daily prednisolone (60 mg/m^2^) until remission, followed by 40 mg/m^2^ every other day for 6 weeks.

10.1136/bmjopen-2018-027011.supp1 Supplementary data

When using study medication, patients will have regular check-ups for monitoring disease activity and adverse events (table 1). Adverse events are defined by any undesirable event that occurs after inclusion until 30 days after stopping study medication.

**Table 1:** Overview of study visits. All assessments at week 0 are part of standard of care

Visit	0	R1	2	3	4	5		6	7	8	9	Relapse	EoS
Week	0	4	8	12	16	20		28	36	44	52		104
Informed consent		X											
Entry criteria	X	X											
Medical history	X	X											
Physical examination	X	X	X	X	X	X		X	X	X	X	X	X
Laboratory examination	X	X	X	X	X	X		X	X	X	X	X	(X)
(Serious) adverse events		X	X	X	X	X		X	X				
Prednisolone													
Study medication													
Quality of life	X	X						X			X		X
Drug dispensing		X			X								
Drug accountability					X			X					

The colour shades indicate in which period prednisolone and study medication is used.

EoS, end of study; R, randomisation; (X), if indicated by treating physician.

### Investigational medicinal product

Levamisole (Elmisol) and placebo tablets are produced, according to Good Manufacturing Practices, and supplied by ACE Pharmaceuticals B.V., Zeewolde, The Netherlands. Elmisol is registered as an orphan drug (European Medicines Agency, 2005). Currently, it is prescribed off-label for the treatment of SSNS in children. Tablets are available in four strengths: 5, 10, 25 and 50 mg. The placebo tablets will match the active drug tablets in appearance, taste and weight, and will only contain pharmacologically inactive substances. Bottles with tablets will be sent to the patient by the Academic Medical Center (AMC) clinical pharmacy.

### Questionnaires

To investigate the effect of INS on the HRQoL, psychosocial adaptation, school performance and parental distress, parents and patients (if 8 years and older) will be asked to complete HRQoL questionnaires at five different time points: at first presentation, at 4 weeks after presentation (prednisolone only), at 28 weeks after presentation (after study treatment), at 1 year after presentation (primary endpoint) and at 2 years after presentation (end of study). Different validated HRQoL questionnaires will be used (table 2).^13–15^ The Paediatric Quality of Life Inventory Generic Scale 4.0 consists of both self and proxy reports including a broad age range (2–18 years).^13^ Norm scores for a Dutch healthy norm population and for children with other chronic illnesses (eg, asthma) are available.^16 17^ The Strength and Difficulties Questionnaire (SDQ) assesses the psychosocial adjustment of children and adolescents.^14 18 19^ Lastly, the Distress Thermometer for Parents is a brief screening instrument to identify distress and everyday problems in parents of children with a chronic condition.^15 20^ In addition, general questionnaires regarding demographics, medication and compliance will be sent to all parents and/or children. KLIK (*Dutch*: Kwaliteit van Leven In Kaart) is a web-based platform for HRQoL questionnaires, developed by the Psychosocial Department of our hospital and used by over 10 000 patients and 400 healthcare professionals.^21^ It is used for both daily clinical care and research in a broad paediatric field. In this study, the KLIK website (www.hetklikt.nu) will be used for filling out the questionnaires; it will only be used for research purposes. In contrast to daily clinical use, the patient will not receive feedback by his or her treating physician. After registration on the website, parents and, if applicable, patients of 8 years and older receive a reminder by email 7 days prior to their next visit.

**Table 2:** Overview of quality of life questionnaires used per age range. At each time point, all questionnaires are provided

Age range	2–3 years	4 years	5–7 years	8–10 years	11–12 years	13–16 years
	Parent about parent	Parent about parent	Parent about parent	Parent about parent	Parent about parent	Parent about parent
Sociodemographics*	General	General	General	General	General	General
Parental distress†	DT-P	DT-P	DT-P	DT-P	DT-P	DT-P

	Parent about child	Parent about child	Parent about child	Parent about child	Parent about child	Parent about child
QOL child	PedsQL (2–4)	PedsQL (2–4)	PedsQL (5–7)			
Psychosocial adjustment	SDQ (2–3)	SDQ (4–18)	SDQ (4–18)	SDQ (4–18)	SDQ (4–18)	SDQ (4–18)
Problems with medication and compliance	Medication and compliance	Medication and compliance	Medication and compliance			
School		School	School			

	Child	Child	Child	Child	Child	Child
QOL child				PedsQL (8–12)	PedsQL (8–12)	PedsQL (13–18)
Psychosocial adjustment					SDQ (11–16)	SDQ (11–16)
Problem with medication and compliance				Medication and compliance	Medication and compliance	Medication and compliance
School				School	School	School

*Once at baseline.

†By both parents, if applicable.

DT-P, Distress Thermometer for Parents; PedsQL, Paediatric Quality of Life Inventory; QOL, quality of life; SDQ, Strengths and Difficulties Questionnaire.

### Study endpoints

The primary endpoint of the study is the occurrence of a first relapse within 12 months (week 52) after first presentation. Relapse is defined as having proteinuria (3+ on urine dipstick confirmed by >200 mg/mmol creatinine on quantitative urinalysis) for three consecutive days.

Secondary endpoints can be found in box 2.

Box 2 **Secondary endpoints are being compared between the intervention groups**

- Time to first relapse (days).
- Relapse rate over 2-year period (n/year).
- Cumulative steroid dose over 2-year period (mg/m^2^).
- Number of (serious) adverse events (n).
- Number of treatment discontinuations (n).
- Proportion patients with FRNS or SDNS (%).
- Days of missing school (days), outpatient visits (n), hospitalisation days (days).
- HRQoL and psychosocial adaptation at different time points (see table 1):
  - Cross-sectional and longitudinal.
  - Comparison with healthy peers and other chronic diseases.

FRNS, frequent relapsing nephrotic syndrome; HRQoL, health-related quality of life; SDNS, steroid-dependent nephrotic syndrome.

### Sample size

Sample size was calculated based on the superiority design of the trial, as well as on the intention-to-treat principle and on the primary outcome of the study: occurrence of relapse within 12 months after first presentation. To achieve a power of 80% with a two-sided significance level of 5%, two groups of 41 subjects are needed to identify a reduction in relapse of 30% (from 75% relapse to 45%), which is considered clinically relevant. A total of 82 patients need to complete 2 years of follow-up. With an estimated drop-out rate of 10%, 92 patients in total need to be included for the RCT. It is expected that around 90% of all children with a first episode of INS will be steroid sensitive.^22 23^ Additionally, we expect that about 65% of the patients screened for eligibility are willing to participate.^4^ In conclusion, this means a total of 157 patients need to be recruited. With an incidence of 60 new cases of INS per year in the Netherlands and 30 new cases per year in Belgium (estimated), an inclusion period of 2 years is expected. After withdrawal of informed consent, no study subjects will be replaced.

### Recruitment

**Figure 2:**
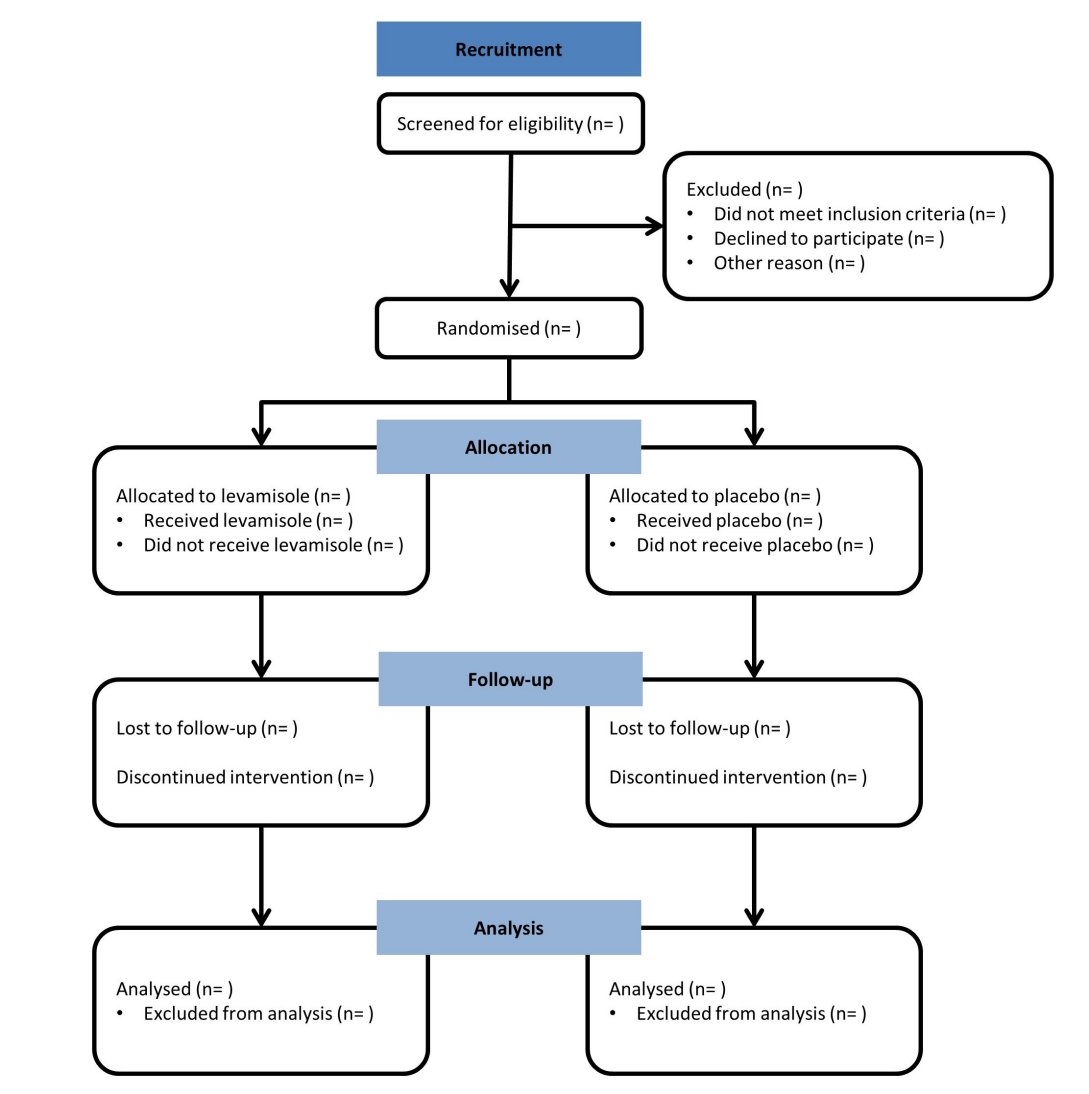
CONSORT flowchart of study design. CONSORT, Consolidated Standards of Reporting Trials.

When patients present in one of the participating hospitals (see online supplement 2) with a first episode of INS, they will be informed about the RCT, after which they receive further written information about the RCT. When a patient presents with a first episode of INS in a non-participating hospital, the coordinating study team is contacted. The treating paediatrician will explain the study briefly and, if patient and parents agree, they will receive further written information by email. If interested in participating in the trial, the patient will be referred to the nearest participating hospital. After 4 weeks, patients will be checked if remission has been achieved. If so, informed consent for the RCT will be obtained and when the patient meets all of the inclusion criteria and none of the exclusion criteria, he/she will be randomised (figure 2).

10.1136/bmjopen-2018-027011.supp2 Supplementary data

To make sure all paediatricians in non-participating centres are acquainted with the trial, a monthly reminder by email will be sent. Also, additional information about the study for paediatricians, general practitioners, parents and patients (as well as all patient information forms (PIFs)) will be available on the study website (www.learns.nl). For questions, they can contact the coordinating investigator.

### Randomisation

A total of 92 eligible patients will be randomly assigned to one of the two treatment arms in a 1:1 ratio, receiving either levamisole (treatment) or placebo (control) added to standard corticosteroid treatment. Block randomisation will occur using block sizes of 2, 4 and 6, which will be randomly used in order to make concealment of allocation as accurate as possible while granting minimal differences in numbers between the two groups. Randomisation (week 4) will be performed by the local investigator using CastorEDC. The local investigator is blinded for the outcome of randomisation; only members of the AMC clinical pharmacy have rights to view allocation of treatment. Patients, parents, medical and nursing staff, outcome assessors, monitors and data analysts are blinded to treatment allocation.

Treatment allocation will be blinded until the study has ended, all data has been collected, queries have been resolved and the database has been locked.

### Data collection and management

Patient data will be collected at every visit by the local investigator and/or coordinating investigator. Clinical data will be entered in the Good Clinical Practice compliant electronic data management system CastorEDC. Additionally, patients are asked to keep a diary on treatment compliance, adverse events, relapse (proteinuria) and days of illness and school absence. These data will also be collected on the electronic case report form. Questionnaires on HRQoL will be completed at home at scheduled time points on the KLIK website (www.hetklikt.nu). After registering, parents receive a reminder to fill out questionnaires.

### Statistical analysis

The outcomes of all included study subjects will be analysed as intention-to-treat. For safety analysis, an ‘as treated’ population will be defined. Study subjects who are lost to follow-up, withdraw informed consent or stopped study medication prematurely will be analysed according to allocated treatment group (figure 2). All data that have been collected until withdrawal or lost to follow-up will be used for analyses. Missing values at baseline and/or follow-up will not be imputed. R Studio will be used for performing all statistical analyses. A p value of <0.05 will be considered statistically significant. Continuous data will be presented as means with SD or median with IQR, depending on distribution. For between group comparisons, a Student’s t-test in case of normal distribution or Mann-Whitney U test in case of non-normal distribution will be used. Categorical data will be presented as proportions and compared using a χ^2^ test. The primary outcome, the occurrence of a relapse at 1 year after first presentation, will be compared between groups using a χ^2^ test. Relapse-free survival will be presented as a Kaplan-Meier survival curve. Multivariate analyses (age at onset, sex, ethnicity, time to remission and FRNS/SDNS) will be performed using Cox regression.

No interim analysis for either efficacy or futility will be performed. Safety reviews will be performed by the Data Safety Monitoring Board (DSMB) after 1 year or when 25 patients have been included and have completed the 24 weeks (week 28 after first presentation) using study medication.

### Monitoring and safety

A risk analysis according to guidelines of the Dutch Federations of University Hospitals categorises the study as ‘moderate risk’. Although the risk of the study is considered negligible, our paediatric study population may be considered vulnerable and, therefore, the risk was upgraded to moderate. The Clinical Research Unit of the AMC will monitor the study, both on site and remotely.

Additionally, a DSMB is established to oversee the inclusion rate and to protect the safety of the study subjects and the integrity of the study data. The DSMB consists of four members: a paediatrician with experience as a member of the ethical committee (chair), a paediatric nephrologist, two methodologists and a nephrologist, all independent of the trial. The DSMB will meet every 6 months, or sooner in case of a suspected unexpected serious adverse reaction (SUSAR). After 1 year or after 25 subjects (whichever occurs first) have been included and completed 24 weeks of treatment with study medication, a safety assessment will be performed. No stopping rules have been defined. The coordinating study team is responsible for reporting serious adverse events and submitting annual safety reports to the central Medical Ethical Committee (MEC) and competent authority. SUSARs will be reported to the competent authority and MEC by ACE Pharmaceuticals in order to keep the study team blinded. The AMC clinical pharmacy will provide unblinding on request after approval by the principal investigator.

### Patient and public involvement

The Dutch Kidney Patient Association (NVN) was involved in the design of the study. Together with the patients, the burden of the intervention was discussed, and, on special request from the NVN, a HRQoL study was implemented in the design. All published data will be made available to the patients through email and the study’s website.

## Ethics and dissemination

### Ethical approval

A notification of no objection has been obtained from the Dutch and Belgian competent authorities: Central Committee on Research Involving Human Subjects (CCMO) and Federal Association of Health and Medicinal Product, respectively. Substantial amendments will be submitted for review to the MECs and competent authorities. The study is registered at the EU Clinical Trials Register under number NL61906.018.17. The study will be conducted in line with the Declaration of Helsinki (2013) and the International Conference of Harmonisation-Good Clinical Practice guidelines.

### Informed consent

Before any study-related (screening) procedures occur, written informed consent must have been obtained from patient’s parents or legal representatives and/or the patient, depending on the patient’s age. Children aged 12 years and older need to sign informed consent in addition to their parents. Children <12 years of age will be informed about the study in a manner that matches their age and understanding. Patients and their parents will be informed about their right to withdraw consent at any time without having to provide reasons. In case of resistance of the patient to any of the study procedures, the ‘*Code of conduct involving minor’* will be used as a guideline. PIFs and informed consent forms are all approved by the MEC.

### Confidentiality

All patients have their own patient ID, generated by the hospital information system. Source data will be stored confidentially at the local sites under this patient’s hospital ID. Study data will be kept using a unique patient identification number. The investigators will keep this number strictly confidential. Only the investigators have access to the key. Handling personal data will comply with the latest General Data Protection Regulation Act (EU Directive, 25 May 2018). All data will be stored for 15 years after the end of the study.

### Dissemination policy

Prior to the start of the study, the LEARNS study was registered in the Dutch Trial Registry. Results will be published in (inter)national peer-reviewed scientific journals once the study is completed. Papers will be published according to the CCMO and International Committee for Medical Journal Editors guidelines. Final results will be made public to all participants.

## Discussion

The LEARNS study aims to demonstrate the efficacy and safety of adding levamisole to standard steroid therapy in children with a first episode of INS on reducing relapses by conducting a multicentre randomised, double-blind, placebo-controlled trial.

Previous research mainly focused on the duration and cumulative doses of prednisolone in children with a first episode of INS. Initially, longer duration of prednisolone treatment was considered superior in order to prevent relapses. Recent, well-conducted studies have shown that treating patients with prednisolone longer (2 or 3 vs 6 months) has no beneficial effects on relapse-free survival.^4 24–26^ Still, in both groups, around 80% of the children experience one or more relapses. In 2006, a study by Hoyer *et al* on the efficacy of cyclosporine as adjuvant to steroid therapy for a first episode of INS showed a significant decrease in relapses in the first 12 months. However, this effect disappeared after 18 months. Because of the high rate of adverse effects and the need for blood monitoring, the authors did not recommend the protocol as standard therapy for these patients.^27^ Currently, the INTENT (Initial treatment of steroid-sensitive idiopathic nephrotic syndrom in children with mycophenolate mofetil versus prednisone) study is ongoing. This open-label, randomised trial compares prednisone versus MMF as initial treatment in children with a first episode of SSNS.^28^ However, apart from these few studies, no further improvement in the prevention of relapses after the first episode of INS has been made.

Relapses of INS consequently result in repeated courses of prednisolone and are often accompanied by steroid toxicity. Therefore, INS comes with a high burden of disease affecting HRQoL of both patients and their families. Although it is known that HRQoL is impaired in children with kidney disease, these studies were mainly performed in children with end-stage renal disease.^29^ Only few studies have been published on HRQoL in children with INS.^30–33^ Not only do these children report a lower HRQoL compared with healthy peers, their parents report an even more pessimistic view.^31^ Additionally, it appears that the duration of the disease has a negative impact on HRQoL.^32^ However, these studies are of cross-sectional design and did not include disease status or medication. Preventing relapses may thus contribute to improving quality of life in INS patients and their families.

Levamisole is a promising steroid-sparing agent since it has relatively few side effects compared with other steroid-sparing drugs. This has been shown in several RCTs in children with FRNS and SDNS.^6–8 12 34–37^ Its mode of action is still poorly understood. It is believed that levamisole has immunomodulatory effects by shifting the Th2 immune response to a Th1 response,^9 10^ and/or has a direct effect on the podocyte by inducing glucocorticoid receptor signalling pathway and, in this way, mimics or enhances the effect of prednisolone.^38^

Furthermore, it must be stressed that in preventing the progression of INS to a chronic illness, research should focus on a permanent cure of the disease. We hypothesise that adding levamisole to the standard treatment with corticosteroids in children with a first episode of INS will prevent future relapses and, thus, will contribute to a better quality of life by reducing the cumulative steroid dose.

Since a similar trial on the efficacy of levamisole is simultaneously conducted in the Paris area, we adapted the French protocol for initial steroid treatment of INS. The study treatment and primary outcome is similar in both studies, but the duration of follow-up is different: 12 months compared with 24 months in our study. Additionally, HRQoL questionnaires were originally not implemented, but Nephrovir-3 has planned to participate in our HRQoL study.

The main strength of our study is its randomised, placebo-controlled design, conducted in 20 participating hospitals in the Netherlands and Belgium. In this way, an extensive, geographical area will be covered. Placebo-controlled RCTs remain the golden standard in comparing different treatment regimens. On the other hand, an important limitation of this study may be the size of the study population. We only powered for our primary outcome, not for subgroup analyses. In case of insufficient inclusions, we may pool our data with the Nephrovir-3 trial since we use similar treatment protocols. Another limitation may be that the treatment of relapses is not standardised by our study protocol as we mainly focus on the initial treatment. However, in the Netherlands, relapses are almost always treated according to national guidelines.^39^

After completion, the LEARNS study may provide evidence for adjusting (inter)national protocols for the standard treatment of the first episode of INS. If adding levamisole would prevent relapses, the disease could be less prevalent and the burden of disease on both patient and society be lowered. This could also mean that repeated courses of corticosteroids will be no longer needed and toxicity will be minimal. Additionally, and not the least important, quality of life of the patients and their families could be improved.

### Trial status

The study started recruiting in April 2018 and is currently recruiting.
