# Supplementary material for: Prevention of relapses with levamisole as adjuvant therapy in children with a first episode of idiopathic nephrotic syndrome: study protocol for a double blind, randomised placebo-controlled trial (the LEARNS study)
Source: BMJ Open. 2019 Aug 1;9(8):e027011. doi: 10.1136/bmjopen-2018-027011 (PMC6688689; doi:10.1136/bmjopen-2018-027011)
Supplement: Supplementary data [file bmjopen-2018-027011supp001.pdf]

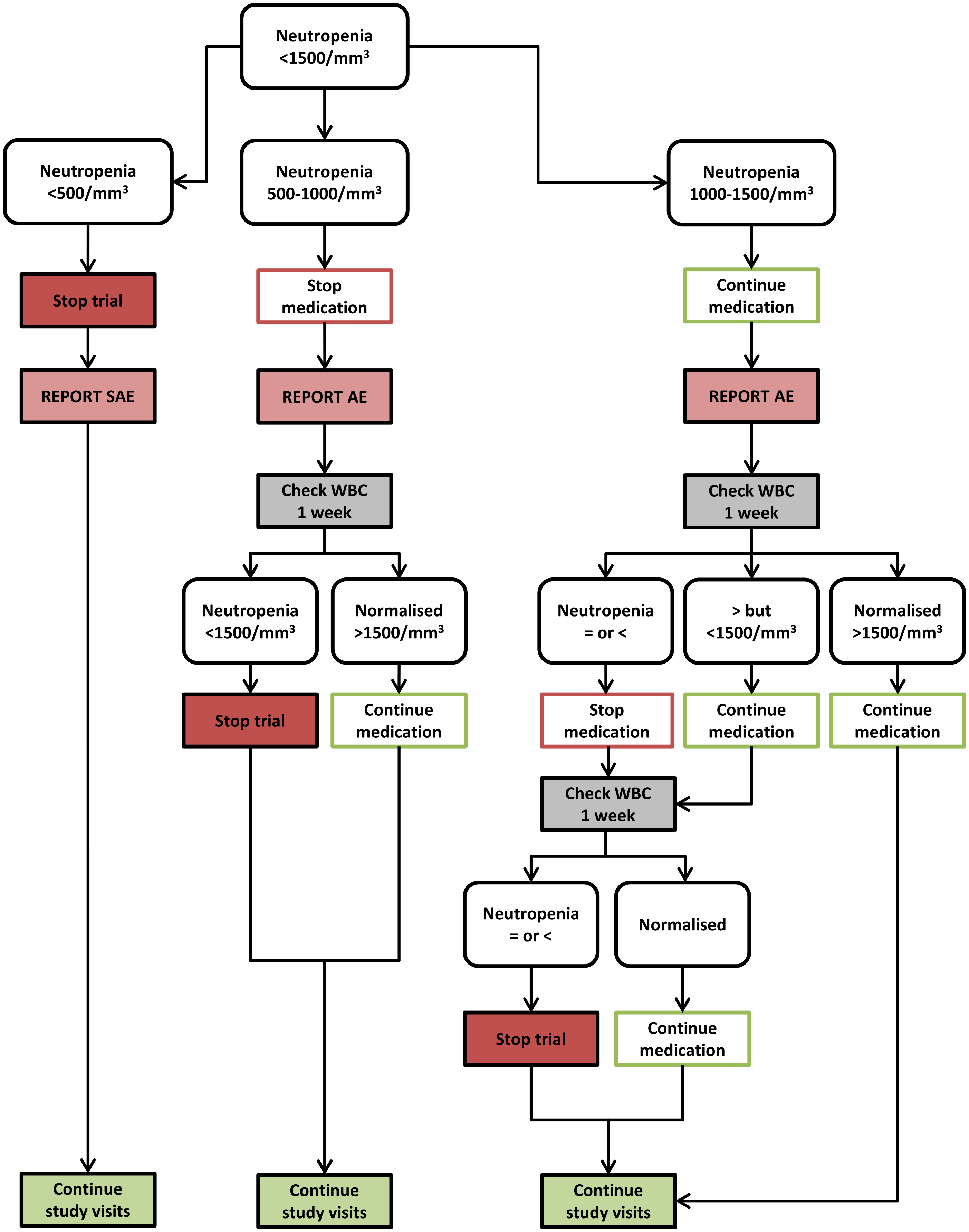


*Supplement 1*. Flowchart of actions regarding the continuation of study treatment in case of neutropenia.
