# Supplementary material for: Prevention of relapses with levamisole as adjuvant therapy in children with a first episode of idiopathic nephrotic syndrome: study protocol for a double blind, randomised placebo-controlled trial (the LEARNS study)
Source: BMJ Open. 2019 Aug 1;9(8):e027011. doi: 10.1136/bmjopen-2018-027011 (PMC6688689; doi:10.1136/bmjopen-2018-027011)
Supplement: Supplementary data [file bmjopen-2018-027011supp002.pdf]

The Netherlands	
**City**	**Hospital name**
Alkmaar	Noordwest Hospital Group
Amsterdam	Emma Children’s Hospital, Amsterdam UMC, location AMC
Amsterdam	Amsterdam UMC, location VUmc
Breda	Amphia Hospital
Deventer	Deventer Hospital
Enschede	Medisch Spectrum Twente
Groningen	Beatrix Children’s Hospital, University Medical Center Groningen
Haarlem	Spaarne Hospital
Leiden	Willem-Alexander Children’s Hospital, Leiden University Medical Center
Maastricht	Maastricht UMC +
Nijmegen	Amalia Children’s Hospital, Radboudumc
Rotterdam	Sophia Children’s Hospital, Erasmus Medical Center
The Hague	Juliana Children’s Hospital, Haga Hospital
Veldhoven	Máxima Medical Center
Zwolle	Isala
**Belgium**	
**City**	**Hospital name**
Antwerp	Queen Paola Children’s Hospital, ZNA
Brussels	Queen Fabiola’s Children’s University Hospital
Ghent	Princess Elisabeth Children’s Hospital, University Hospital Ghent
Leuven	University Hospitals Leuven
Liège	Clinique d’Ésperance, University Hospital Center Liège

Supplement 1 **List of study sites participating in the LEARNS study**
